# Toward characterizing extracellular vesicles at a single-particle level

**DOI:** 10.1186/s12929-019-0502-4

**Published:** 2019-01-15

**Authors:** Chun-yi Chiang, Chihchen Chen

**Affiliations:** 1 Institute of Nanoengineering and Microsystems, National Tsing Hua University, Hsinchu, 30013 Taiwan; 2 Department of Power Mechanical Engineering, National Tsing Hua University, Hsinchu, Taiwan

**Keywords:** Extracellular vesicle, Single particle, Exosome, Microvesicle, Electrophoresis, Raman spectroscopy, Atomic force microscopy, Digital PCR

## Abstract

Extracellular vesicles (EVs) are cell-derived membrane-bound vesicles that serve a means of cell-cell communication. Studying EVs at a single-particle level is important because EVs are inherently heterogeneous. Novel micro- and nanotechnological tools have open opportunities for realizing single-EV measurements exploiting their biochemical, electrical, mechanical, and/or optical properties. This review summarizes the recent development of technologies toward sorting and analyzing single EVs. Sorting EVs into a more homogeneous subset relaxes the sensitivity and throughput required on the EV detection, and hence related techniques are also included in this review. These exciting technologies are on the rise and will expand our understanding of EVs and their applications in the near future.

## Background

Extracellular vesicles (EVs) are double-layered membrane vesicles derived from most cells and released into biological fluids [1–3]. EVs are 30–5000 nm in diameter and contain biological molecules, including mRNAs, miRNAs, lipids, and proteins. EVs play key roles in both physiological and pathological processes [4–6] and have been known as one of the mediators in cancer metastasis [7, 8]. Surface proteins as well as nucleic acids of EVs show potential to serve as biomarkers of many diseases, such as cardiovascular diseases, parasite infections, tumor metastasis and tumor angiogenesis [9, 10]. However, EVs are heterogeneous in size and composition, as such; their biophysical properties, such as refractive index and density, also vary [10, 11]. The heterogeneity is in part due to the status of their parental cell, the diversity of cargo and the existence of several biogenesis routes [12]. It is of great importance to be able to probe EVs at a single-particle level to fully reveal their biological functions and clinical potentials. Currently, this is still quite a challenging task, partly due to the limited detection sensitivity and contaminating particles, such as cellular debris, exomeres, lipoproteins, protein aggregates, and virions [13, 14]. In this review, the recent development of techniques toward sorting and detecting EVs at a single-particle level is introduced. Together, these techniques, when matured, will allow us to obtain EVs or subsets of EVs rid of contaminants and provide statistically valid information that is often difficult, if not impossible, to obtain by measuring large ensembles of EVs.

## Extracellular vesicles (EVs): Properties and a brief history of their discovery

The presence of EVs in blood was implicated and originally reported in 1946 by Chargaff and West [15]. EVs were later referred to as “platelet dust” by Wolf in 1967 [16]. In the 1970–1980s, EV had been observed as plasma membrane fragments released from rectal adenoma microvillus cells [17]. Around the same time, the significantly stronger immunoreactivity of tumor-originated membrane fragments was demonstrated [18]. In 1983, detailed studies showed that vesicles are also released upon fusion of multi-vesicular bodies (MVBs) with the plasma membrane [19]. Later, Raposo and colleagues demonstrated that these vesicles, then termed exosomes, isolated from virus-transformed B lymphocytes, were antigen-presenting and able to induce T cell responses [20]. In 2007, with the discovery that EVs contain RNA, including microRNA, EVs acquired substantially renewed interest as mediators of cell-to-cell communication [4].

EVs are heterogeneous membranous vesicles and have been isolated from most cell types and biological fluids such as saliva, urine, nasal and bronchial lavage fluid, amniotic fluid, breast milk, plasma, serum and seminal fluid [21]. Based on their size and presumed biogenetic pathways, EVs have been currently defined into three main subgroups: apoptotic bodies, microvesicles, and exosomes [22]. Apoptotic bodies, 50–5000 nm in diameter, are released during apoptosis when plasma membrane blebbing occurs. Microvesicles, 100–1000 nm in diameter, are produced by budding and pinching off from the plasma membrane directly. Exosomes, smaller than 100 nm, originate from the endocytic compartment [23, 24]. Although these subgroups of vesicles are of distinct biogenesis routes, they are of overlapped physical and chemical properties. Conventional sample preparations often yield a mixed population of vesicles, and hence they are collectively termed extracellular vesicles.

EVs are composed of proteins, lipids, and nucleic acids that are derived from the parental cell [25, 26]. The nucleic acids including DNAs, coding and non-coding RNAs, such as mRNAs and microRNAs (miRNAs) [4]. It has been shown that transferring EVs is accompanied with re-programming of the recipient cell activities and functions [27]. Proteomic studies show many EVs contain proteins that are common among all EVs regardless of the types of parental cells, whereas only a small fraction of proteins are cell-specific, reflecting the type and (patho)physiological conditions of those secreting cells [23]. Some specific proteomic characteristics have been proposed for the subgroups of EVs, but there are still not widely accepted specific markers to distinguish them yet.

The heterogeneity of EVs has been clearly demonstrated recently. Much different morphology of EVs in bodily fluid samples have been observed using cryogenic electron microscopy (cryoEM) [28]. In human ejaculate, about 59% of EVs are found to be single vesicles, while the rest are oval vesicles, double vesicles, double special vesicles, triple vesicles, tubules, lamellar bodies, etc. [28]. The morphological variability of EVs suggests the existence of different subpopulations which may possess different functions and biochemistry. Recognizing the high heterogeneity of EVs, it is imperative to sort them into respective populations in order to comprehend their contents and roles in physiological and pathological processes. However, the tools for analysis of EVs of different intracellular origins, and thus probably different functions are still under development. In the interim, carefully determined contaminants in pre-analytical treatment conditions should be considered respectively for sorting and characterizing EVs from different biological fluids. EVs derived from non-sterile body fluids, such as nasal fluid, saliva and milk may contain bacteria-derived material [29]. In addition, biofluid-specific contaminants, such as Tamm-Horsfall glycoprotein in urine as well as glycosaminoglycans and proteoglycans in synovial fluid samples should be eliminated before protein/saccharide-related characterization [30]. Evaluation focusing on not only the presence of the selected markers but also the absence of contaminants is recommended. For EV-RNA analysis, plasma is the most commonly used source of EVs; therefore the protein-RNA complexes such as Argonaute (AGO) proteins [31] and lipoproteins such as low-density lipoprotein (LDL) and high-density lipoprotein (HDL) should be considered [32]. Currently, a multidimensional EV purification strategy is often employed to obtain highly-purified EVs or EV subgroups for the subsequent profiling of EV cargo. In doing so, more insight and properties in the composition and function of specific EVs are acquired, and EV-based biomarkers are to be identified.

## EV isolation techniques

For sorting EV subpopulations, five main groups of EV sorting techniques have been developed, including differential ultracentrifugation (DUC)-based techniques, size-based techniques, immunoaffinity capture-based techniques, polymer-based precipitation, and microfluidic techniques.

Ultracentrifugation-based techniques are the most commonly used and reported techniques for EV isolation. During differential ultracentrifugation (DUC) procedures, the sample is subjected to a centrifugal force, and particulates are sedimented sequentially according to their density, size, and shape. The pellet is re-suspended in an appropriate medium, while the supernatant is subjected to subsequent runs of centrifugation with increasing centrifugal force. Hence, subpopulations of EVs are pelleted and sorted at different runs of centrifugation [33].

The pelleting time (*T*) can be predicted using Eq. 1,

1 $$ T=k/s $$

where *k* is the clearing factor of the rotor, or *k* factor, and *s* is the sedimentation coefficient. Therefore the pelleting time depends on the settings of the centrifuge, the physical properties of the particulates, as well as the viscosity of the solvent. This approach requires minimal additional reagents, sample pretreatments, and technical expertise. However, EV pellets obtained are often contaminated with protein aggregates, lipoproteins, and other particles when body fluids are processed. Density gradient ultracentrifugation can be conducted after ultracentrifugation to remove contaminants that differ in density, such as protein aggregates. This approach is considered the “gold standard” for EV isolation [30]. However, density gradient ultracentrifugation is time consuming (62–90 h) in processing complicated biological samples [34] and requires costly equipment (around $50–100 k) [35–37]. Both make simultaneously processing a large number of samples not feasible for standard hospital laboratories and resource-poor settings [35]. In addition, low EV yield (5–25% recovery) [38], centrifuge-induced deterioration of EV integrity, and lipoprotein contaminant make this method challenging for clinical applications. DUC protocols may also induce aggregation of EVs in highly concentrated suspensions. In addition, repeated freeze and thaw cycles may disrupt the integrity of EVs [39] and change their biological activity [40]. It is suggested adding 25 mM trehalose may reduce aggregation of EVs during ultracentrifugation protocols and conserve the integrity of EVs during freezing and thawing cycles [39].

Size-based techniques, such as ultrafiltration and size exclusion chromatography (SEC), sort EVs based on their size. Ultrafiltration utilizes a membrane of defined sized pores that allow small particles to pass through, but retain large particles in the concentrate. Ultrafiltration is faster than ultracentrifugation and does not require special equipment and additional reagent. However, protein contamination and poor biological activities are anticipated due to the shear-force-induced deformation and breakup of large vesicles. In addition, EVs loss due to attaching to the membrane may potentially deviate the results of downstream analysis [41]. Size exclusion chromatography (SEC) is another size-based separation technique applied to EV sorting. In SEC, a porous stationary phase is utilized to sort macromolecules and particulate matters out according to their size. Components in a sample with small hydrodynamic radii are able to enter the stationary phase, thus resulting in late elution. On the contrary, larger components are excluded and remain in the mobile phase, thus getting eluted earlier. The mobile phase is typically driven by gravity, albeit the longer process time, in order to preserve the integrity and bioactivity of EVs.

Immunoaffinity capture-based techniques utilize capture molecule-conjugated substrate or magnetic beads to pull down EVs that harbor target molecules on their surface. Captured EVs may be subsequently recovered using respective elution solution. EVs have been reported with the presence of various membrane biomarkers. A good biomarker for immunoisolation needs to be membrane-bound, lacking soluble counterparts, and solely expressed or highly concentrated on the surface of EVs from specific biological sources. The immunoaffinity capture approach with much smaller sample volumes has produced comparable results to those obtained by ultracentrifugation. It may be more effective than ultracentrifugation given the availability, specificity, and affinity between the capture molecule and EV surface marker [42].

EVs can be settled out of biological fluids by altering their solubility or dispersibility via adding polymers, such as polyethylene glycol (PEG). This method is originally commonly used to isolate viruses. EV precipitate can be easily pelleted under low-speed centrifugation. Therefore, polymer precipitation is easy to use and does not require any specialized equipment. This allows its easy integration into clinical usage and is scalable for large sample sizes [41]. However, many contaminants, such as proteins and precipitating polymers, are often co-isolated [34]. Pre- and post-isolation steps are employed to reduce these contaminants if required. The pre-isolation step often involves the removal of subcellular particles, such as lipoproteins. The post-isolation step is typically employed to remove the polymer by using a desalting column, such as Sephadex G-25 [34].

The fast advance in microfabrication technology has offered an exciting opportunity for the fabrication of microfluidic-based devices to sort EVs rapidly and efficiently, basing on both physical and biochemical properties of EVs at the microscale. For clinical uses, inventions of microfluidic methods for EVs sorting and detection provide a new approach for EV characterization. These methods require smaller amounts of samples and are generally faster and more sensitive than traditional technologies. Microfluidic immune-affinity approaches for EV trapping have been demonstrated [43–45]. The quality and quantity of RNA extracted from trapped EVs are sufficient for downstream polymerase chain reaction (PCR) or microarray analysis. However, the immune-affinity approach enriches only a subpopulation of EVs with a specific surface protein [35]. Microfluidic devices incorporated with porous polymer sieves are capable of collecting EVs without immuno-selectivity. Typical issues associated with filtration, such as pore clogging, EV trapping, and contamination may be lessened by driving the filtration by electrophoresis instead of pressure. Microfluidic devices allow a much lower voltage to be used due to their small size [46]. Wang et al. have demonstrated size-based trapping of liposomes using ciliated nanowire-on-micropillar hierarchical structures [47]. Trapped particles can be released by dissolving silicon nanowires in phosphate-buffered saline (PBS) overnight. Asymmetric flow field-flow fractionation (AF4) technology has been used widely for sorting and characterizing nanoparticles, polymers, proteins, and recently EVs [14, 48]. In AF4, analytes are first introduced into a flat channel by a laminar tangential flow, and then a transverse flow is applied to sort analytes based on their diffusion coefficients. Most isolation methods, however, still require additional off-chip steps, such as sample preparation, nucleic acid extraction, and quantification.

## EV characterization methods

As more researchers have been committed to developing high-throughput methods for accurate EV subpopulations sorting and characterization, more microfluidic devices are designed to integrate with different techniques allowing for better EV separation and detection. Im et al. have developed a nano-plasmonic exosome (nPLEX) sensor, which consists of periodic nanoholes patterned in a gold film [49]. Binding of EVs to the vicinity of nanoholes increases the refractive index, which causes spectral shift and the intensity changes of the transmitted light. The observed limit of detection is ~ 3000 EVs, which corresponds to a sensitivity that is four orders of magnitude higher than western blot and two orders of magnitude higher than chemiluminescence enzyme-linked immunosorbent assay (ELISA). Moreover, this method enables the continuous and real-time monitoring of the molecular binding event without labeling.

Methods for characterizing extracellular vesicles at a single-particle have been reported in recent years, and more are on the way. In this review, three categories of methods are introduced and summarised in Table 1, including 1) optical methods: nanoparticle tracking analysis (NTA), dark-field microscopy, flow cytometry, and laser tweezers Raman spectroscopy (LTRS), 2) non-optical methods: transmission electron microscopy (TEM), cryo-EM, atomic force microscopy, and impedance-based detection, and 3) digital methods for measuring biochemical compositions.

**Table 1:** Single EV characterization techniques

Technique	Detection principle	Size range	Concentration range (particle/mL)	Throughput (particle /min)
Optical methods				
Nanoparticle tracking analysis	Brownian motion, scattered and fluorescent lights	50 nm − 1 μm	10^7^–10^9^	6000
Dark-field microscopy	Scattered light	> 50 nm	relative	–
Flow cytometry	Scattered and fluorescent lights	20 nm −40 μm	10^7^–10^10^	10,000
Laser tweezers Raman spectroscopy	Inelastically scattered light	20 nm −100 μm	relative	0.2
Non-optical methods				
Electron microscopy	Scattered electrons	1 nm −10 μm	10^10^–10^12^	–
Atomic force microscopy	Interaction forces between the probing tip and the sample	1 nm	relative	–
Impedance-based flow cytometry	Coulter principle	50 nm −10 μm	10^5^–10^12^	3000
Digital methods				
Digital PCR	PCR in partitions	–	< 200	200
Digital ELISA	ELISA in partitions	–	< 10^4^	1500

### Optical methods

When a particle, e.g. an EV, is under light irradiation, how light is scattered depends on the wavelength of the incident light (*λ*) and properties of the particle, including its shape, diameter (*d*), relative refractive index to the ambient medium (*m*), and absorption coefficient. When the EV diameter is greater than one-tenth of the wavelength, the intensity of light scattered is proportional to the fourth power of the diameter as predicted by Mie theory [50]. However, when EVs are at least 10 times smaller than the wavelength, the Rayleigh approximation predicts the intensity of light scattered (*I*) is proportional to the sixth power of the diameter as described in Eq. 2,

2 $$ \boldsymbol{I}\propto \frac{{\boldsymbol{d}}^{\mathbf{6}}}{{\boldsymbol{\lambda}}^{\mathbf{4}}}{\left(\frac{{\boldsymbol{m}}^{\mathbf{2}}-\mathbf{1}}{{\boldsymbol{m}}^{\mathbf{2}}+\mathbf{2}}\right)}^{\mathbf{2}} $$

where ∝ indicates “proportional to” [51, 52]. Therefore, a relatively small difference in diameter will result in a large difference in the light scattered. For example, a 40-nm EV scatters more than ten times brighter than a 27-nm one of the same refractive index. An ensemble of methods has been utilized to characterize EVs based on the detection of scattered light, including dynamic light scattering (DLS), nanoparticle tracking analysis (NTA) and flow cytometry [53]. The DLS method utilizes the time-scale fluctuation of scattered light to determine the diffusion coefficient and hence the size of particles. Although DLS is capable of measuring particles ranging from 1 nm to 6 μm, it does not measure individual particles, and hence it is suitable for detecting monodispersed particles and is less exact in characterizing vesicles of heterogeneous size distributions [54].

#### Nanoparticle tracking analysis (NTA)

Similar to DLS, NTA depends on tracking the Brownian motion of particles in suspension to infer their size; but unlike DLS, NTA is an image-based method. It consists of a laser module, a microscope, a sensitive charge-coupled device (CCD) camera, and a fluidic chamber. The scattering signals from individual particles within the field of view were tracked and video-recorded. An accurate evaluation of the size profile requires long track lengths, steady temperature and viscosity, and a proper dilution of samples. NTA is able to measure particles of 10^7^–10^9^/mL in concentration, which corresponds to approximately 1–80 particles in the field of view, which is about 100 μm × 80 μm × 10 μm in standard NTA measurements. However, in practice, EV concentration in the range of 2 × 10^8^ - 20 × 10^8^/mL has been recommended [55]. Too few EVs in the field of view introduces statistical sampling errors, while too many results in overlapped scattering signals by neighboring EVs, especially when polydisperse samples are measured. EVs as small as 50 nm in size can be detected. The sample preparation for NTA is minimum and the sample may be easily recovered after the measurement. However, NTA does not discriminate between EVs and other particles. One solution is to fluorescently label EV markers. However, it is challenging since only a fraction of EVs may carry the target marker, which is often present at low copy numbers. In addition, signals may be interfered by free dye molecules or dye aggregates, especially in the case of small EVs [54].

#### Dark-field microscopy

**Fig. 1:**
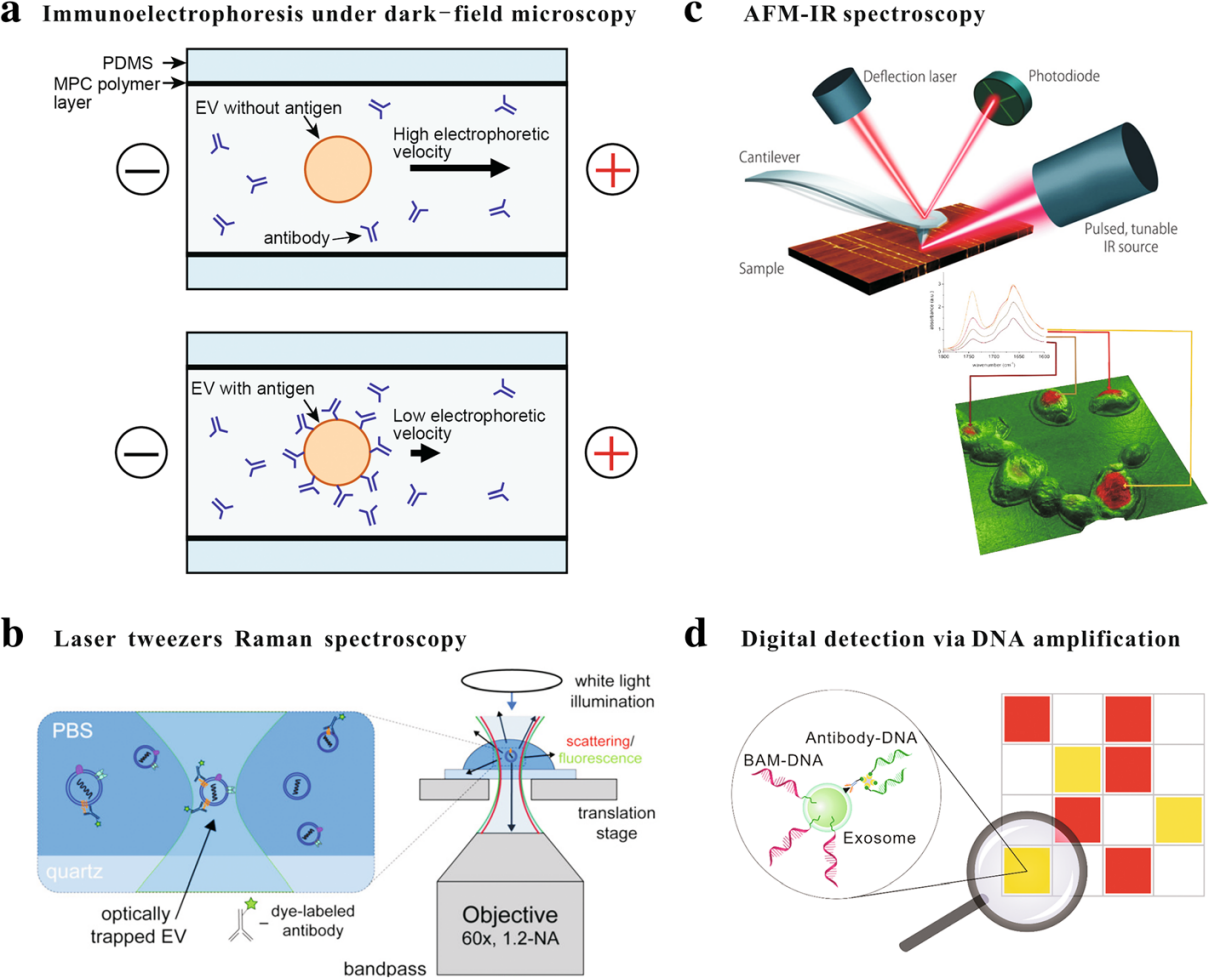
Emerging techniques for probing single extracellular vesicles. **a** EVs are driven electrophoretically inside a microchannel toward the anode. The microchannel is made of poly(dimethylsiloxane) (PDMS) and coated with a phospholipid copolymer containing 2-methacryloyloxyethyl phosphorylcholine (MPC) and 3-methacryloxyethyl triethoxysilane (METESi) to suppress electroosmotic flow and nonspecific adsorption. The movement of EVs, visualized under dark-field microscopy, may change its speed upon binding of antibodies [57]. **b** Schematic of multispectral optical tweezers which allows for the simultaneous measurement of fluorescence and Raman spectra on trapped EVs [75]. **c** Schematic diagram of AFM-IR. The AFM tip detects the local IR absorption of the sample excited by a pulsed tunable laser source [89]. **d** EVs labeled with biocompatible anchor molecule (BAM)-DNA or antibody-DNA conjugates are randomly distributed into microfluidic chambers. Nuclei acid-based amplification gives digitalized signals from each chamber, indicating the presence of EVs or specific target molecules [92]. Images reprinted with permissions

Dark-field microscopy collects only light scattered by the sample, and hence the image is usually of a decent signal-to-noise (S/N) ratio. Dark-field microscopy has been integrated with on-chip microcapillary electrophoresis to assess the zeta potential of individual EVs [56]. The motion of individual EVs can be visualized in a dark field by detecting the scattered laser light, and the mobility shift of EVs upon immunolabeling can be used to profile the biochemical compositions of EVs as shown schematically in Fig. 1(a) [57]. It has been shown that the distribution of the zeta potential of untreated EVs is symmetric with a mean of − 10.2 mV, and it becomes skewed toward − 3.4 mV when EVs are labeled with positively charged antibodies [57].

#### Flow cytometry

Conventional flow cytometry measures physical properties and internal complexity characteristics of single cells or EVs above 500 nm in diameter by collecting scattered light at different angles. Physical properties such as the size of particles can be evaluated by forward-scattered light (FSC) with a scatter angle between 0.5–5°, while the internal complexity such as granularity of internal structures can be assessed by side-scattered light (SSC) with a scatter angle ranging from 15° to 150° [58]. EVs below 500 nm produce scatter light in the range of the electronic noise; therefore forward scatter is not feasible to resolve this size range [59]. Furthermore, forward scatter may be variable between instruments of different manufacturers [54]. Side-scattered light is often collected at the 90° angle and has a better sensitivity than FSC to provide information on smaller particles near 190 nm in diameter [60]. The multiple-angle forward scattering method, which measures FSC at multiple angles, offers an improved resolution for detecting smaller microparticles [61]. Fluorescent dyes have been used for profiling EV components, such as proteins and nucleic acids. After incubating EVs with specific labels, a density gradient ultracentrifugation may be performed to rid the sample of free dye molecules and aggregates. Detecting EVs stained with fluorescent dyes, such as PKH26 lipophilic dye, is also a method used for improving the S/N ratio that is independent of the size and refractive index of EVs [62]. Many advantages of characterizing EV by flow cytometer have been demonstrated, such as high-throughput measurements, evaluation and quantification of the surface protein [63]. However, the coincident event detection, or swarm effects, confines the detection concentration range. Swarm effect occurs when two or more particles simultaneously arrive at the measuring spot, and a measurement derived from these multiple particles is misidentified as a single event by the flow cytometer. With the increase of event rates, the presence of a permanent scatter at the measuring spot eventually arises, resulting in the incapability of the flow cytometer to distinguish events. Event rates drop hereafter along with overestimated scatter signals that mostly occur in the detection of high concentrations of EVs [64]. Furthermore, the refractive index of calibration standards should be considered to assure the accuracy of comparing EV diameter. Despite the limitation of size in EV analysis using flow cytometer, many modified methods have been reported. For instance, latex beads coated with antibodies have been utilized to enrich specific EV populations of interest, permitting the bulk analysis of EVs below 100 nm in diameter. Parametric information of specific EV subpopulations has also been performed by flow cytometer [65]. Nano-scale flow cytometric sorting (nanoFACS) using high sensitivity multiparametric scattered light and fluorescence measurements is an emerging method for analyzing and sorting individual EVs as well as other nanoscale particles, such as liposomes and viruses [66]. Single EVs larger than 100 nm can be characterized by conducting fluorescence labeling, size exclusion chromatography (SEC), and nanoFACS subsequently. Analysis of single EVs down to 40 nm using high-sensitivity flow cytometer has been demonstrated [67].

#### Laser tweezers Raman spectroscopy (LTRS)

Raman spectroscopy is one of the molecular scattering-based methods capable of detecting chemical properties and chemical dynamics toward a single cell or organelle level [68, 69]. When monochromatic incident radiation strikes at a sample and interacts with sample molecules, scattered radiation at all directions arises. Much of the scattered radiation has a frequency same as the incident radiation, which constitutes Rayleigh scattering. A small fraction of the scattered radiation is of a different frequency due to the inelastic collision between the incident monochromatic radiation and molecules of the sample, which constitutes Raman scattering [70]. Raman scattering can be used for both qualitative and quantitative purposes. The frequency and intensity of scattered radiations reveal the quality and quantity of the sample, respectively [71]. It has been successfully applied to many biomedical topics, such as cancer detection [72], orthopedic surveillances [73], and drug of abuse evaluations [74]. LTRS is one form of Raman spectroscopy, in which a tightly focused laser beam is utilized to trap the particle and also as the incident light as schematically shown in Fig. 1(b) [75]. LTRS with a confocal detection setup allows collecting Raman signals from only the focal volume, which makes it possible to detect subcellular particles such as lipid droplets [76] and EVs [77]. LTRS can be integrated with vibrational or fluorescence spectroscopy to provide an even more comprehensive characterization of single EVs [75]. The non-contact and label-free nature of LTRS empowers it for time-course analysis [78]. However, the response time and throughput do not yet meet requirements for profiling applications. The weak Raman signal, typically 1 million times weaker than fluorescence labeling, results in a long integration time. For example, LTRS may take around 5 min to obtain a spectrum from a single EV [77].

### Non-optical methods

In microscopy, the achievable resolution is given by the Rayleigh criterion as shown in Eq. 3,

3 $$ \boldsymbol{R}=\frac{\mathbf{1.22}\boldsymbol{\lambda }}{\mathbf{2}\boldsymbol{NA}} $$

where *R* means the resolution, *λ* is the wavelength, and *NA* is the numerical aperture of the microscope objective. To attain a resolution beyond what visible light can provide, two approaches have been developed, namely, electron microscopy (EM) and atomic force microscopy (AFM). Impedance-based flow cytometry will also be discussed.

#### Electron microscopy (EM)

In electron microscopy, a beam of electron is emitted, accelerated and focused onto the specimen in a vacuum environment. As the wavelength of electrons is more than 10^3^ shorter than that of visible light, electron microscopy can achieve a resolution of 1 nm. The higher the acceleration voltage, the shorter the wavelength, and hence the better the resolution, but also more damaging to biological samples. Among the various EM techniques, transmission electron microscopy (TEM) and cryo-electron microscopy (cryo-EM) have been commonly used for EV characterizations [79]. In both techniques, an image is created by collecting electrons transmitted through an ultrathin specimen. The thickness of the sample is typically less than 100 nm for TEM and 500 nm for cryo-EM. To increase the contrast of biological samples under EM, the fastest way to prepare a negatively stained EV sample, in which a layer of heavy metal salts covers the specimen [80, 81]. Combining EM with immunogold labeling makes it possible to gain biochemical information [79]. However, it usually takes many hours to prepare samples for TEM, and the fixation and dehydration in the procedures often affect the size and morphology of EVs [51]. In contrast, cryo-EM images on rapid freezing samples at a very low temperature, reducing the sample damaging and artifacts caused by the addition of heavy metals, dehydration or fixation steps at a cost of a lower contrast [80]. Polymorphism of EVs derived from even a single cell type has been clearly revealed under cryo-EM [80]. Freezing samples rapidly is critical to prevent the formation of ordered crystalline ice; therefore the native structure of the sample is preserved [82]. Cryo-EM has been used to visualize membrane bilayers and internal features of single EVs [83, 84]. Functionalized gold nanoparticles can be applied as fiducial markers to probe biochemical compositions of EVs [80].

#### Atomic force microscopy (AFM)

Atomic force microscopy is a powerful method for studying samples in nanoscale and was developed by Binnig’s group in the 1980s [85]. The operation principle of AFM is detecting and recording interactions between the probing tip and the sample surface. The AFM probe consists of a cantilever with a sharp tip mounted at the free end. The deflection of the cantilever caused by interaction forces is recorded by a laser and a position sensitive detector. The lateral resolution of ~ 3 nm and the vertical resolution of below 0.1 nm achievable to AFM make it well suited for probing surface profiles of EVs [85]. In AFM, EVs must be bound to an extremely flat surface, such as mica for measurement. Successful AFM imaging of EVs in air and in liquid have been reported. When imaged in air, EVs often appear flatter and softer in the center, presenting a typical “cup shape” [86]. In comparison, EVs retain their native spherical shape when imaged under in-liquid conditions [87]. Substructures of EVs of around 1 nm resolution have been demonstrated by using ultra-sensitive low-force AFM [86]. In additional to probing mechanical properties of EVs, the tip and/or the substrate of the AFM can be further modified with molecules such as antibodies to examine their biochemical characteristics. Compared with immunogold labeling in EM, it is possible to recognize surface compositions of EVs using AFM with a better resolution in a near-native environment [88]. Atomic force microscopy-based infrared spectroscopy (AFM-IR), which utilizes the AFM tip to detect localized thermal expansion in a sample excited by a focused laser pulse, is an emerging technique that can provide simultaneous chemical, mechanical analyses and imaging capabilities with a very fine spatial resolution (Fig. 1(c)) [89].

#### Impedance-based flow cytometry

Impedance-based flow cytometry works based on the Wallace Coulter principle that is able to resolve EVs that are larger than 50 nm. This technique relies on a current pulse generated when a non-conducting particle suspending in the electrolyte passes through a pore where each particle displaces its own volume of the electrolyte solution and increases the impedance [90]. Hence, the magnitude of the current pulse is essentially proportional to the volume of the particle. In addition, the frequency of current pulses reflects the particle concentration, and the duration of the current pulse can be used to assess the surface charges carried by the particle. Therefore, this technique is capable of simultaneously determining the surface charge, concentration, and size distribution of EVs. It is relatively fast, real-time, label-free, viscosity-independent, and does not require large sample volumes. However, pore clogging can occur with large and/or too many particles.

### Digital methods

Digital detection, such as digital PCR, provides unique advantages for performing single-molecule detection [91]. In digital detection, targets are randomly segregated into partitions. Signals from as little as a single molecule are amplified in individual partitions and counted digitally at the reaction endpoint [92–94]. Absolute quantification of rare targets is possible and with an increased S/N ratio. Tian et al. exploited well-established nucleic acid assays on EV quantification [92, 93]. EVs are first tagged with DNA oligonucleotides, which can be achieved via anchor molecules or antibodies. Labeled EVs are then distributed into microfluidic chambers. Subsequent nucleic acid amplification, such as PCR or rapid isothermal nucleic acid detection assay (RIDA), reveals the presence/absence of EVs or specific surface molecules inside each chamber. The starting concentration of EVs can be inferred based on a Poison distribution. Quantitative analysis and detection of EVs at a single-particle level have been demonstrated [95].

## Conclusions

Cellular heterogeneity is a fundamental principle of cell biology. Our understanding of the behavior of cells has been greatly advanced by analyzing single cells in large numbers. Similarly, the functions of EVs may be attributed to an ensemble of EVs or only a few dominating rare EVs. The heterogeneity and relatively small size of EVs pose great challenges in their characterization and applications. Currently, an optimal EV isolation technique is yet to be developed. Differential ultracentrifugation (DUC) provides reproducible sorting product and is currently mostly used for research purpose, but not yet regularly used clinically due to low yields and time-consuming procedures of this method. Using DUC in a non-urgent situation, such as the cancer assessment and diagnosis could be feasible. Microfluidic-based methods offer advantages in high throughput, cost-effective diagnostics using a small amount of sample. They are suitable for an emergency situation, such as myocardial diseases. However, more biological validation and reproducibility need to be established. A combination of techniques, such as SEC-DUC, enriches more homogeneous EV subpopulations. Toward characterizing extracellular vesicles at a single-particle level benefits from hybrid approaches using two or more sorting techniques to refined target EVs subpopulations, and suitable characterization methods depend on the research purpose to achieve biological validation and specifically reproducibility. The demands of clinical applications such as low cost, reliability, high resolution, and throughput may eventually be met with modifications of technologies for improved quantifiability and measurability.

## Abbreviations

AF4: Asymmetric flow field-flow fractionation
AFM: Atomic force microscopy
AFM-IR: Atomic force microscopy-based infrared spectroscopy
AGO: Argonaute
BAM: Biocompatible anchor molecule
CCD: Charge-coupled device
cryo-EM: Cryo-electron microscopy
DLS: Dynamic light scattering
DNA: Deoxyribonucleic acid
DUC: Differential ultracentrifugation
ELISA: Enzyme-linked immunosorbent assay
EV: Extracellular vesicle
FSC: Forward scattered
HDL: High-density lipoprotein
LDL: Low-density lipoprotein
LTRS: Laser tweezers Raman spectroscopy
METESi: 3-methacryloxyethyl trimethoxysilane
miRNA: microRNA
MPC: 2-methacryloyloxyethyl phosphorylcholine
mRNA: Messenger RNA
MVB: Multi-vesicular body
nanoFACS: Nano-scale flow cytometric sorting
nPLEX: Nano plasmonic exosome sensor
NTA: Nanoparticle tracking analysis
PCR: Polymerase chain reaction
PDMS: Poly(dimethylsiloxane)
RIDA: Rapid isothermal nucleic acid detection assay
RNA: Ribonucleic acid
S/N: Signal-to-noise
SEC: Size exclusion chromatography
SPR: Surface plasmon resonance
SSC: Side scattered
TEM: Transmission electron microscopy
